# Mexican Rice Borer Control Tactics in United States Sugarcane

**DOI:** 10.3390/insects10060160

**Published:** 2019-06-05

**Authors:** Allan T. Showler

**Affiliations:** Knipling-Bushland U.S. Livestock Insects Research Laboratory, USDA-ARS, 2700 Fredericksburg Road, Kerrville, TX 78028, USA; allan.showler@ars.usda.gov, Tel.: (830) 792-0341

**Keywords:** biological control, cultivar, cultural control, irrigation, predators, physiochemicals, insecticides, IPM, maize, natural enemies, nitrogen, parasitoids, resistance, soil, transgenic, trap crop, varieties, water deficit

## Abstract

The invasive Mexican rice borer, Eoreuma loftini (Dyar), expanded its range from Mexico to South Texas in the early 1980s. By 2008 the pest had moved into sugarcane- and rice-growing areas of East Texas and Louisiana, and by 2012 it was reported on noncrop host plants in Florida. Efforts to suppress E. loftini in United States sugarcane with chemicals and biological control agents were unsuccessful, so both tactics were discontinued, and E. loftini infestation of sugarcane has continued unchecked. During the last 15 years, however, research has focused on the pest’s ecology, improved insecticides and scouting methods, the identification of sugarcane resistance mechanisms, and new cultural tactics. A surveillance technique was developed that indicates when larvae are most vulnerable to insecticide sprays. Currently, registered insecticides for E. loftini control are not widely applied, although some show promise, including an insect growth regulator. A number of potentially useful cultural practices are available, including plowing under fallow stubble, judicious use of fertilizer, adequate irrigation, avoiding proximity to E. loftini-susceptible maize cultivars, and enhancement of natural enemy populations. Demonstrated and potentially useful sugarcane resistance mechanisms involve physiochemical attributes, physical characteristics, and transgenic cultivars.

## 1. Introduction

The Mexican rice borer, *Eoreuma loftini* (Dyar) (Lepidoptera: Crambidae), is indigenous to western Mexico, where it is a major pest of sugarcane, *Saccharum* spp. [1,2]. The moth was first detected in the United States in Texas, 1980 [3,4,5]. By 1989 it was infesting rice fields of east Texas [6,7,8]. The pest was found in Louisiana rice in 2008 [9,10,11,12], and by early 2015 *E. loftini* had spread to rice and sugarcane in eight southern Louisiana parishes [13]. In 2012 and 2013, adult and immature *E*. *loftini* were found on nonsugarcane host plants in two Florida counties [14,15,16].

The pest usually oviposits 5 to >80 eggs per female in clusters within folds of dry, and sometimes green, sugarcane leaves [17]. *Eoreuma loftini* prefers to deposit eggs on senescing foliage and on water deficit-stressed stressed plants [18]. Early instars feed on leaves, beneath leaf sheaths; others tunnel into the leaf midrib, and later instars bore into the main stalk [19]. Economic injury occurs to mature stalks by diminishing sugar yield, and by stunting and lodging stalks occasionally so severely that heavily afflicted fields are not harvested [9,10,11,12,13,14,15,16,17,18,19,20,21,22]. Although tunneling mostly occurs in lower internodes, younger internodes are also vulnerable, including those near and at the plant’s apex [16]. Tunnels are oriented horizontally and vertically, and they are packed with frass, protecting larvae from insecticides and natural enemies [9]. The larval stage can last as long as 33–53 d [23,24], after which mature larvae pupate inside the stalk for 7–10 d [25]. The straw-colored adults, delta-shaped without markings, emerge through an exit hole adjacent to the pupa [16]. In the subtropics, where the insect occurs in the United States, a life cycle lasts 30–45 d, and 4–6 overlapping generations occur each year [20,22]. The lower development threshold for *E. loftini* is 13.9 °C, and the approximate degree days required for development of eggs, larvae, and pupae are 87.5, 249.3, and 122, respectively [23,24]. In northern Tamaulipas, Mexico, half of overwintering *E*. *loftini* in maize emerge during February, and populations are relatively high in June and July with corresponding levels of damage [16,20]. Where sugarcane and maize, *Zea mays* L., is grown, harvest of maize fields in late summer forces movement into sugarcane, which remains in the field for 2–4 more months [26].

*Eoreuma loftini* injures 20% of sugarcane internodes in South Texas, incidentally providing portals for red rot, *Colletotrichum falcatum* Went., which breaks down sugar [20,27,28]. Larval population densities do not usually exceed one larva per sugarcane stalk in South Texas [29] but 50%–80% bored internodes were reported on some cultivars in Texas [3,4,30]. Nineteen percent bored internode injury in Texas resulted in an estimated decline in sugar yield of ≈25% [31]. Infestations of Texas sugarcane cost US$575–$690 per hectare [19,21], amounting to US$10–20 million in annual losses [22,31]. Economic impacts for Louisiana include US$220 million and $45 million in projected losses to sugarcane and rice production, respectively [8]. *Eoreuma loftini* attacks ≥17 species of graminous weedy host plants and ≥6 crop species including rice, *Oryzae sativa* L.; sugarcane; maize; and sorghum, *Sorghum bicolor* (L.) Moench [16,32]. 

## 2. Monitoring

Control of pests often depends on measuring populations through field sampling. A sex pheromone placed in a Universal Moth Trap (Unitrap, Great Lakes IPM, Vestaburg, MI) is used for monitoring adult *E*. *loftini* populations [13,19,33]. Other methods for population estimation are time-consuming and labor-intensive: sweep netting, stalk inspections, and stalk dissection [33]. Wilson et al. [19] determined that the percentage of stalks with larvae exposed on plant surfaces is proportional to the abundance of moths captured in pheromone traps; an intervention threshold of 20 moths/trap/week indicates the presence of >5% infested stalks. When novaluron, a chitin synthesis inhibitor, was sprayed using a 5% infested stalk threshold in South Texas, a single application was required for protection during one season [19]. *Eoreuma loftini* larvae are typically exposed 1–2 d on the surface of the plant, and this can be extended on resistant varieties [19] such that thresholds for chemical intervention against *E*. *loftini* might be adjusted in order to better target larvae when they are most vulnerable [16].

## 3. Insecticides

Insecticide use against *E. loftini* in South Texas was discontinued during the 1990s [22] because applications were not associated with improved yields [21,31]. Relatively recent field tests demonstrate that some insecticides (i.e., tebufenozide, flubendiamide, beta cyfluthrin, and chlorantraniliprole [a seed treatment registered for use in rice and sugarcane]) decreased percentages of bored internodes 3- to 8.5-fold [34,35]. Novaluron is an insect growth regulator that is more effective than many other insecticides because of its longer efficacy under field conditions and enhanced trans-laminar dispersion within the plant, and because of its less toxic effects on beneficial arthropods [19]. A single application of novaluron suppressed *E*. *loftini* injury within economically acceptable levels while heightening sugar production by 14% in commercial fields [19], but this result has been inconsistent. Although some promising insecticides are registered for use against *E*. *loftini*, their application is still not prevalent.

## 4. Biological Control

Because *E. loftini* blocks its larval tunnels with frass, natural enemy activity against the pest is impeded [22]. The red imported fire ant, *Solenopsis invicta* Buren, which is an effective predator against the sugarcane borer, *Diatraea saccharalis* (F.) [36], thrives in moist environments [37,38] but it is much less common in dry South Texas [39]. A preliminary study by VanWeelden et al. [40] in the wetter environment of East Texas reported that exit holes of *E. loftini* were 44.4% less abundant in sugarcane where red imported fire ants were not suppressed with pesticides. 

Nine parasitoids indigenous to the Lower Rio Grande Valley and 18 exotic species were released in the field between 1982 and 1997 for *E. loftini* control [16,41,42]. Some of the parasitoid species were not recovered after they were released [43]. The best rate of parasitism on larvae and pupae averaged ≈6.2% by indigenous braconids *Chelonus sonorensis* Cameron and *Digonogastra solitaria* (Wharton and Quicke) and by two exotic species, *Alabagrus stigma* (Brullé) and *Allorhogas pyralophagus* (Marsh) [44]. In Mexico, only 6.9% of *E*. *loftini* larvae were parasitized by the native tachinid Jalisco fly, *Lydella jalisco* Woodley [45]. Introduced to South Texas [6], most parasitism by the Jalisco fly occurred in late summer and early fall, reaching ≈30%, after which populations diminished [46]. Suppression of *E. loftini* in the United States using parasitoids and parasites has been weak in spite of numerous attempts [16,42,44,47]. Entomopathogens, including *Nosema pyrausta* Paillot and *Beauveria bassiana*, and nematodes *Steinernema riobravis* Cabanillas, Pinar and Raulston, *S. carpocapsas* (Weiser), and *S. feltae* Filipjev have shown some promise in laboratory trials, but efficacy in the field, largely owing to desiccation and poor contact with the target pest, has been weak [22,42,46,48].

## 5. Fallow and Planting Date

Sugarcane stubble in fallow fields that are plowed into the soil can reduce overwintering *E. loftini* larvae, and planting pest-free seed pieces improves the development of setts (clumps of stalks growing from the same buried stalk node) [49]. Although early planting has shown some efficacy against *D. saccharalis* [50], planting dates have not been evaluated for their effects on *E. loftini* in sugarcane.

## 6. Irrigation

*Eoreuma loftini* tends to oviposit on sugarcane plants that are water deficit-stressed [16,18,51,52]. Sugarcane cultivar L 03-371, for example, is resistant to *E*. *loftini* under wet conditions but it is susceptible when conditions are dry (up to 88% bored internodes) [30,53]. Well-irrigated sugarcane was 1.8-fold less vulnerable to *E. loftini* oviposition, and injury to stalks was reduced by ≈2.5-fold compared to drought stressed sugarcane in both susceptible (LC 85-384) and resistant (HoCP 85-845) cultivars [52]. Oviposition preference and levels of injury to stalks relative to each cultivar, however, did not change (i.e., HoCP 85-845 had 1.8-fold less injury than LCP 85-384 within each irrigation regime) [51,52]. In the greenhouse, well-watered sugarcane plants had 82.8% to 90.2% fewer eggs on the stalks and leaves than water deficit-stressed plants. Numbers of larval entry holes on stalks ranged from 44.4% to 94.5% fewer, and adult exit holes were 64.3% to 88.9% less abundant [18]. Well-irrigated sugarcane had 78.7% fewer dry leaves, *E. loftini*’s preferred oviposition substrate, than drought stressed sugarcane [18]. Living sugarcane leaves on water deficit-stressed plants might also be more attractive than on nonstressed plants, because of heightened accumulations of nutrients such as free amino acids that are essential for insect growth and development [18,51]. Studies have demonstrated that heightened concentrations of essential (and nonessential) free amino acids in sugarcane are associated with drought stress [18,51,52]. In regions that are subject to dry conditions or drought, sugarcane should be kept adequately irrigated to avoid exacerbating *E. loftini* infestations [16,51,54,55].

## 7. Preharvest Leaf Removal

Burning leaves on sugarcane just prior to harvest, a routine practice in some places, kills insect eggs on them, but the effect on later life stages is probably relatively small because larvae and pupae are not present in dry leaf tissue (most are inside the stalk), and adults can avoid the flames by flying away from the flames. The impact of burning leaves against *E. loftini* populations before harvest has not been investigated, but even supposing that substantial numbers of *E. loftini* were eliminated, protection of the crop at harvest would be too late to be of value. Sometimes, sugarcane leaves are instead stripped from the stalks, occasionally cut into small pieces, and deposited as a 15–20-metric ton/ha mat, 8–10 cm deep [56,57], of leaf material, or “greenchop”, on the soil surface [58]. Harvested sugarcane stalks in South Texas during the planting season had 2.3- and 2.8-fold more *E. loftini* entry holes per stalk in field plots where greenchop was left on the soil surface as mulch and where greenchop was mechanically incorporated 20 cm deep into the soil, respectively, than in plots without greenchop (ATS, unpublished data). Such effects, however, were not observed during the ratoon (regrowth from previous year) season. 

## 8. Nitrogen Application 

Applying large amounts of nitrogen 8 mo before planting can increase soil nitrate-N several-fold over amounts common to nonamended soils [59]. Sugarcane leaf tissue grown on high nitrogen soil had significantly more (≥14.3%) percentage N than leaf tissue grown on low nitrogen and on nonamended soils during the planting season and ≥1.2% in the first ratoon [59]. The leaves from plants grown on high nitrogen soil were found to have more of three essential and several nonessential free amino acids, and more fructose, than leaves grown on low nitrogen and nonamended soils [59]. Fructose concentrations in *E. loftini* host plant species indicate that fructose is a limiting nutritional factor [60]. Entry holes in sugarcane stalks were ≥2.3-fold more abundant when the crop was grown on high nitrogen soil than on low nitrogen and nonamended soils, and exit holes ≥2 times more abundant [59]. While sugarcane stools growing in high nitrogen soil averaged 18% more stalks in the first growing season than in low nitrogen and nonamended soils, the increase was offset by intensified *E. loftini* injury (stalks in the low nitrogen and nonamended soils were 16% and 31% heavier, respectively) [59]. Judicious application of nitrogenous fertilizer has been recommended for avoiding heavy *E. loftini* infestations [16,59].

## 9. Proximity to *E. loftini*-Susceptible Maize

*Eoreuma loftini* prefers susceptible maize cultivars over sugarcane; the maize “traps” the pest until it is harvested and the adults fly into sugarcane [32,41]. In South Texas, 3 mo after the late summer maize harvest, entry holes in winter-harvested sugarcane stalks increased by 7- to 8-fold [41]. Reducing movement of *E. loftini* from maize into sugarcane will entail not planting *E. loftini*-susceptible maize cultivars near sugarcane and planting *E. loftini*-resistant maize varieties instead [60].

## 10. Natural Enemy Conservation

Weed growth has been reported to increase herbivorous arthropod populations in sugarcane fields that serve as prey for predators [36,61]. Enhancement of *S. invicta* populations and other natural enemies by permitting limited weed growth in Louisiana sugarcane substantially reduced *D. saccharalis* injury to the crop [36,61]. Preliminary observations suggest that *S. invicta* in nonarid regions can attack *E. loftini* to some extent [40]. Allowing weeds to grow in noncompetitive stands within sugarcane fields might support sufficiently substantial *S. invicta* populations to suppress *E. loftini* abundances in the crop [16]. Where prevalent grass weeds host relatively large *E. loftini* numbers and natural enemies associated with the presence of the weeds do not exert adequate control, the weeds should be expunged [16]. 

Selection of pesticides in sugarcane should be conducted carefully to protect natural enemy populations and consequently to reduce pest incidence [16]. Aldicarb, for example, has sometimes been used to control root knot nematodes, *Meloidogyne* spp., but it strongly diminishes natural enemy populations, including *S. invicta*, and *D. saccharalis* injury to the crop increased by 19.4% to 32.9%, including where weeds were being used to augment natural enemy diversity and abundance [36]. While natural enemies might not be as effective against *E. loftini* as they are against *D. saccharalis*, growers should try to use selective pesticides whenever possible to conserve *S. invicta* colonies and other predators and parasitoids. 

## 11. Cultivar Resistance

Host plant preference is an antixenotic form of resistance that, with regard to *E. loftini*, is characterized by selection of oviposition sites [17,18,32,52,62]. Antibiotic resistance, which decreases survival of one or more of the pest’s life stages, has also been determined for protecting sugarcane against stalk borers [63]. Preference is often governed by the host plant’s nutritional value to the pest [18,52,60]. Wilson et al. [30] developed a “relative resistance ratio” that permits the categorization of sugarcane cultivar resistance based on relative levels of pest preference (antixenosis) and host suitability (antibiosis) although application of relative resistance ratios can be further refined [30]. Resistance in some cultivars is conferred when *E. loftini* larvae remain on the exterior of the plant longer than on other cultivars, which exposes the larvae to natural enemies and pesticides [30].

Some *E. loftini*-resistant sugarcane cultivars have been identified; for example, 1.6-fold more eggs were laid on susceptible LCP 85-384, which produced up to 5.6-fold more adults and had an 18.2% lower recoverable sugar yield than on HoCP 85-845 [51,64]. Additionally, *E. loftini* was responsible for as much as 2.6-fold bored internodes in HoCP 91-555 stalks than were found in HoCP 85-845 stalks [64]. Further, HoCP 00-950 and HoCP 05-902 are susceptible to *E. loftini,* and L 01-299, HoCP 04-838, and HoCP 96-540 are moderately resistant [51,53,64]. The commercial sugarcane cultivar HoCP 85-485 has been reported to be resistant to *E*. *loftini* in three of four small plot field experiments, in contrast with the consistently susceptible HoCP 04-838 cultivar [30]. TCP 87-3388 has also been categorized as *E. loftini*-resistant [30]. The causes of the observed resistance for such cultivars, however, have not been determined. Although some cultivars are moderately resistant to both *E*. *loftini* and *D*. *saccharalis* (e.g., HoCP 04-838) [30,65], other cultivars are resistant to one of the stalkboring species but not to the other. Relatively recent reports have described different mechanisms that confer or might confer resistance to sugarcane through physiochemical and physical characteristics, and potentially through transgenic manipulation [17,66].

### 11.1. Physiochemical

Some sugarcane cultivars that are susceptible to *E. loftini* have ≥2-fold concentrations of certain essential and nonessential free amino acids than resistant varieties, and cultivars of lesser nutritional quality (antixenosis resistance) [67,68]. It is possible that drought-tolerant cultivars might accumulate fewer nutrients associated with water deficit stress, such that the plant would be less attractive for *E. loftini* oviposition and of lesser value for larval development than other, more susceptible, cultivars [18]. Accumulations of free amino acids and fructose in sugarcane have been associated with relatively high *E. loftini* infestations (18,41,51,59]; therefore, breeding sugarcane for low concentrations of those (and possibly other) limiting factor nutrients might provide resistance to *E. loftini* [16,51]. 

### 11.2. Physical

Resistant varieties can lengthen the time larvae are exposed on external plant surfaces before tunneling into the plant, making them increasingly vulnerable to insecticides and biological control agents [19]. Physical factors, including stalk fiber content, leaf sheath appression [69], and rind hardness [70], can each impede larval establishment [30]. US 93-15, a high-fiber cultivar, was among the most resistant of 25 varieties assessed in one study [19], but high fiber content is often associated with relatively poor sugarcane yield [71] and planting such fiber cultivars might not be economically advisable [30].

Number of eggs per sugarcane plant is positively associated with abundances of dry leaves, which typically curl or fold at the edges [17,51]. When provided with a choice between sugarcane leaves that were green and folded, and flat and dry leaves, and with folded paper of the same color as dry sugarcane leaves, eggs were almost exclusively deposited on the folded green leaves [17]. Given a choice between dry folded leaves and folded green leaves, *E. loftini* deposited eggs almost exclusively on the dry folded leaves [17]. In a no-choice cage assay conducted in a greenhouse, potted sugarcane plants with dry leaves (the sugarcane plants also had green leaves) that were trimmed to eliminate tight folds along edges had only 6.2% of *E. loftini* egg deposition on them compared against nontrimmed plants with excised dry sugarcane leaves scattered as a “mulch” on the floor of the greenhouse cage, and against control plants that were not trimmed or mulched [17]. The lack of dry leaf folds on the trimmed plants is an example of horizontal resistance, because of the diminished availability of egg deposition sites and increased risk of egg and neonate mortality absent protection from insecticides and natural enemies within folds [16]. Breeding sugarcane cultivars for leaves that do not fold on drying will likely provide substantial, possibly complete, resistance against *E*. *loftini* that will be difficult to overcome because resistance is conferred by >1 factor. 

### 11.3. Transgenic

Mixed with a meridic diet to obtain a concentration of 0.47% total protein, sugarcane leaf sheath expressing snowdrop lectin (galanthus nivalis agglutinin) did not adversely affect first-generation *E*. *loftini* larval and pupal development and survivorship, fecundity, and egg viability [72]. Snowdrop lectin, however, reduced larval survival, adult emergence, and fecundity in the second generation [72]. No further study of snowdrop lectin has been reported regarding *E*. *loftini* control in sugarcane.

Although transgenic sugarcane is not yet available for control of *E*. *loftini*, the technology can be transferred to sugarcane once its utility is demonstrated in other crops. *Bt* transgenic maize cultivars (registered for protection against numerous lepidopteran pests but not *E*. *loftini*) were tested against non-*Bt* maize varieties for *E. loftini* resistance in South Texas [66]. Maize plants too damaged by *E. loftini* to be harvested were ≥2.5-fold more abundant in two non-*Bt* maize varieties than in transgenic Pioneer 31G71, and ≥18.4 times more abundant than in transgenic Golden Acres 28V81 [66]. The two transgenic varieties were protected from stalk lodging, shattering, and decay that was caused by *E*. *loftini* in the two non-transgenic varieties [66]. Abundances of internodes per maize plant that had been tunneled by *E*. *loftini* were 41.2% and 99% lower in Pioneer 31G71 and in Golden Acres 28V81, respectively, than in the non-*Bt* varieties [66]. Larval entry holes were 49.2% and 99% less abundant in Pioneer 31G71 and in Golden Acres 28V81, respectively [66]. Further, adult exit holes were 93% less numerous in Pioneer 31G71, and adult exit holes were not found on Golden Acres 28V81 stalks [66]. Reared on meridic diet mixed with 5000 µg of Golden Acres 28V81 leaf tissue per ml of diet, 4-wk-old *E*. *loftini* larval weight was 87% lower than larvae maintained on diet with Pioneer 31G71 leaf tissue, and 93% lower than larvae reared on diet mixed with non-*Bt* maize leaf tissue [66].

Because Pioneer 31G71 curtailed <70% of adult emergence, it was only moderately effective as a trap crop for *E*. *loftini*. Ideal “dead end” trap cultivars would be at least as attractive to *E. loftini* compared with susceptible varieties, and the stalks would be tolerant to shattering, lodging, and stalk rot diseases to extend their utility while suppressing adult emergence [16]. Golden Acres 28V81 was nearly totally resistant against larval *E*. *loftini* tunneling [66]. Light-colored larval feeding scars were observed on the outermost tissue of Golden Acres 28V81 without penetration of the stalk, indicative of antibiotic resistance [66]. Golden Acres 28V81 is therefore more suitable as a trap plant for *E*. *loftini* eggs than Pioneer 31G71 [66]. Growing highly biocidal transgenic maize like Golden Acres 28V81 within agricultural landscapes that are partly comprised of sugarcane fields might be a valuable way of “deploying” maize production as a dead end trap crop to protect sugarcane from *E. loftini* [16]. It has been recommended that growers plant sugarcane as far from maize as possible, but the role of transgenics in stalk borer management might supersede that practice [16]. Resistance management, however, could remain an issue [73].

Perhaps a worthy application of the VT3Pro gene in Golden Acres 28V81 (and other efficacious genes) will involve its insertioninto sugarcane [66]. Transgenic sugarcane varieties might offer an especially potent crop protection tactic where *E*. *loftini* is a serious pest [66]. Although some stalkborers, including *E*. *loftini*, might become resistant to *Bt* maize (or, hypothetically, *Bt* sugarcane) cultivars [74], the level of resistance reported in Golden Acres 28V81 against *E*. *loftini* [66] suggests that the VT3Pro gene inserted into sugarcane might provide nearly complete protection against the pest and that use of Golden Acres 28V81 maize (or a similarly effective transgenic variety) as a trap crop can provide partial control [66]. 

## 12. Conclusions

The invasiveness of *E. loftini* in the United States and the ubiquity of host crops and suitable large-stemmed grassy weeds have enabled the pest’s range to range expand from South Texas to a currently limited area of Florida where it might move into sugarcane, a major commercial commodity of that region. Following *E. loftini*’s discovery in the United States in the early 1980s, control measures failed and *E. loftini* attacked South Texas sugarcane unchecked. Relatively recent research (i.e., during the last 15 years), however, has made a rational scouting approach available for timing insecticide applications, developed an array of improved insecticides, and discovered ecological aspects of the pest that can be manipulated to suppress *E. loftini* by adopting cultural practices, as well as physical, physiochemical, and transgenic bases for developing resistant cultivars. Suppression of *E. loftini* populations below economic injury levels in sugarcane will most likely be accomplished by wielding a combination of tactics in an integrated pest management approach capitalizing on resistant cultivars and avoidance of exacerbating field conditions (e.g., drought stress, unnecessary soil pesticides, over-fertilizing, and planting close to susceptible maize varieties). The pest’s mobility and its interaction with other crops in agriculturally diversified regions necessitate the application of such a strategy on an area-wide scale.
